# Exercise‐induced out‐of‐proportion increase in afterload and impaired right ventricular contractile reserve in HFpEF

**DOI:** 10.1002/ehf2.70007

**Published:** 2025-11-10

**Authors:** Jan Sebastian Wolter, Alexander Schulz, Torben Lange, Steffen D. Kriechbaum, Shelby Kutty, Johannes T. Kowallick, Julia M. Treiber, Andreas Rolf, Samuel Sossalla, Gerd Hasenfuß, Andreas Schuster, Sören J. Backhaus

**Affiliations:** ^1^ Department of Cardiology Justus‐Liebig‐University Giessen, Kerckhoff‐Clinic Bad Nauheim Germany; ^2^ German Center for Cardiovascular Research (DZHK) Partner Site Rhine‐Main Bad Nauheim Germany; ^3^ University Medical Center Göttingen, Department of Cardiology and Pneumology Georg‐August University Göttingen Germany; ^4^ German Center for Cardiovascular Research (DZHK) Partner Site Lower Saxony Göttingen Germany; ^5^ Department of Medicine, Cardiovascular Division Beth Israel Deaconess Medical Center and Harvard Medical School Boston Massachusetts USA; ^6^ Taussig Heart Center Johns Hopkins Hospital Baltimore Maryland USA; ^7^ FORUM Radiology Rosdorf Germany; ^8^ Department of Cardiology and Angiology, Medical Clinic I University Hospital Giessen, Justus‐Liebig‐University Giessen Giessen Germany; ^9^ FORUM Cardiology Rosdorf Germany

**Keywords:** afterload, contractility, exercise‐stress, HFpEF, right ventricle

## Abstract

**Aims:**

The pathophysiology of heart failure with preserved ejection fraction (HFpEF) includes pulmonary vascular remodelling and right ventricular (RV) involvement. We sought to investigate the significance of non‐invasive cardiovascular magnetic resonance (CMR)‐derived RV loading conditions.

**Methods:**

Patients with exertional dyspnoea and diastolic dysfunction [E/e′ > 8, left ventricular ejection fraction (LVEF) >50%] underwent rest and exercise‐stress echocardiography, right heart catheterization and CMR. HFpEF was defined by pulmonary capillary wedge pressure [rest ≥15 mmHg (overt) or stress ≥25 mmHg (masked)]; otherwise, patients were classified as non‐cardiac dyspnoea (NCD). CMR‐derived RV haemodynamic indices were defined as follows: afterload Ea = end‐systolic pressure (ESP)/stroke volume (SV), contractility Ees = ESP/left ventricular end‐systolic volume and RV/pulmonary artery coupling as Ea/Ees.

**Results:**

HFpEF (*n* = 34; female 73.5%; median age 69 years) patients showed increased afterload and contractility at rest (Ea 1.20 vs. 0.85, *P* = 0.001, Ees 0.61 vs. 0.37, *P* < 0.001) and during exercise (Ea 2.48 vs. 1.53, Ees 1.00 vs. 0.74, *P* < 0.001) compared with NCD (*n* = 34; female 55.9%; median age 66 years). The relative increase of contractility from rest to stress was smallest in overt HFpEF (overt 1.40 vs. masked 1.86, *P* = 0.001) and highest in NCD (HFpEF 1.56 vs. NCD 1.97, *P* = 0.022). The out‐of‐proportion increase in afterload over contractility in HFpEF was reflected in a statistical trend towards increased Ea/Ees from rest to stress in HFpEF (*P* = 0.078) while Ea/Ees decreased in NCD (*P* = 0.002). Patients with resting Ea or Ees above the median showed lower exercise‐induced increases in cardiac index (Ea: below: 2.8 vs. above: 2.2, *P* = 0.031; Ees: below: 2.9, above: 2.1, *P* < 0.001).

**Conclusions:**

Resting RV afterload elevation in HFpEF results in a compensatory increase in contractility. Out‐of‐proportion increase of afterload paralleled by inadequate increase in contractility results in failure to increase the cardiac index in HFpEF, potentially associated with exertional functional failure.

## Graphical abstract

Non‐cardiac dyspnoea (*n* = 34, NCD) and heart failure with preserved ejection fraction (*n* = 34, HFpEF) patients were classified according to right heart catheterisation. Cardiovascular magnetic resonance derived right ventricular (RV) haemodynamic indices for afterload (Ea), contractility (Ees) and RV/pulmonary artery coupling (Ea/Ees) were evaluated. At rest, RV afterload elevation in HFpEF results in a compensatory increase in contractility. During exercise‐stress an out‐of‐proportion increase of afterload paralleled by inadequate increase in contractility results in failure to increase the cardiac index in HFpEF but not in NCD.

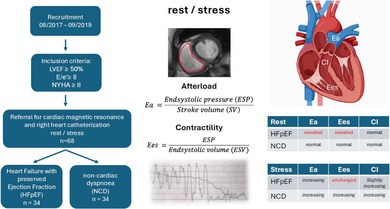

## Introduction

Heart failure (HF) with preserved ejection fraction (HFpEF) had previously been regarded as a disease confined to the left ventricle (LV) characterized by LV stiffening and consecutive diastolic dysfunction.^1^ However, it is now recognized as a heterogeneous syndrome united by elevated left atrial (LA) pressure at rest or during exercise.^2^ Post‐capillary pulmonary hypertension (PH) in turn drives pulmonary vascular remodelling^3^, ^4^, ^5^ and right ventricular (RV) dysfunction.^6^ Emerging from observations of adverse outcomes following interatrial shunt device implantations in the Reduce‐LAP‐II trial,^7^, ^8^ the concept of latent pulmonary vascular disease (PVD) in HFpEF has been introduced and defined by elevated pulmonary vascular resistance (PVR) during exercise‐stress.^9^, ^10^, ^11^ Indeed, due to prognostic implications in PH, the diagnostic threshold of PVR had recently been lowered by the European Society of Cardiology (ESC) from 3 to 2 Wood units (WU).^5^

RV function is load‐dependent, and arterio‐ventricular coupling can provide additional information on RV dysfunction.^12^ Guazzi et al. demonstrated that the relationship of tricuspid annular plane systolic excursion (TAPSE) and pulmonary artery systolic pressure (PASP—a surrogate for the force generated by the RV) is independently of their constituents associated with outcome in HF.^12^ Notwithstanding, available evidence also indicates interdependence of RV strain and afterload as well as ventriculo‐arterial coupling.^13^ However, to date, most studies examining RV function in HFpEF were conducted at rest only.^6^ Limited data indicate impaired RV functional reserve during exercise‐stress.^8^

Cardiac magnetic resonance (CMR) is the reference standard for RV functional evaluation.^13^ In addition to the intricate anatomy of the RV and volumetric assessments, CMR feature tracking (FT) enables the detection of subtle alterations in RV function.^14^, ^15^, ^16^, ^17^, ^18^, ^19^, ^20^ Previously, the HFpEF stress trial demonstrated an association of latent PVD and impaired RV functional reserve during exercise‐stress.^11^ Consequently, we sought to investigate the relationship of RV loading conditions to enhance our pathophysiological comprehension of these conditions in HFpEF.

## Methods

The HFpEF stress study (DZHK‐17, NCT03260621), a single‐centre investigator‐initiated clinical study at the University of Göttingen, prospectively enrolled 75 patients with exertional dyspnoea [New York Heart Association (NYHA) Class ≥II] and echocardiographic evidence of diastolic dysfunction [E/e′ > 8, left ventricular ejection fraction (LVEF) >50%]. Exclusion criteria have been reported previously.^21^ Briefly, patients with coronary artery disease ≥50% stenosis, ≥moderate valvular disease, pulmonary disease and contraindications for CMR were excluded.^21^

All patients underwent rest and exercise‐stress echocardiography, right heart catheterization (RHC) and CMR in the supine position. RHC and echocardiography were performed simultaneously, CMR imaging was conducted 1 day apart, except for one case with a two‐day interval. Data acquisition for exercise‐stress was performed 3 min after reaching a steady‐state heart rate of 100–110 bpm at 50–60 rpm on a bicycle ergometer. All patients were at stable sinus rhythm at the time of imaging. The number of patients with a history of paroxysmal atrial fibrillation is reported in *Table* 1 while patients with permanent atrial fibrillation were excluded. Echocardiography included apical two‐, three‐ and four‐chamber views and parasternal short‐ and long‐axis orientations for colour, pulsed‐wave and continuous‐wave Doppler assessments. E/e′ was measured in lateral and septal positions on apical four‐chamber view. RHC was performed using a Swan‐Ganz catheter placed through the right internal jugular vein for mean cardiac pressure assessments in respective positions of the right atrium (RA), RV and pulmonary artery (PA) including wedge position for measurement of pulmonary capillary wedge pressure (PCWP). Cardiac output (CO) was assessed by the means of thermodilution and based on at least three valid independent measurements over several respiratory cycles and is given as cardiac index (CI) indexed to body surface area (BSA). PVR was calculated using the following formula $\mathrm{PVR}=\frac{\text{PAPm}-\text{PCWP}}{\mathrm{CO}}$ where PAPm is mean PA pressure.

**Table 1:** Patients characteristics.

Variable	HFpEF *n* = 34	Non cardiac dyspnoea *n* = 34	Significance *P*
Age (years)	69 (67, 77)	66 (52, 73)	**0.034**
Sex male/female	9/25	15/19	0.128
NYHA classification	II *n* = 21	II *n* = 27	0.110
	III *n* = 13	III *n* = 7	
Atrial fibrillation	16	5	**0.004**
H2FPEF score	5.0 (3.0, 6.3)	3.0 (2.0, 5.0)	**0.003**
HFA‐PEFF score	5.5 (3.8, 6.0)	4.0 (2.0, 4.0)	**<0.001**
Cardiovascular risk factors			
Active smoking	4	5	0.720
Hypertension	27	27	1.000
Hyperlipoproteinemia	21	21	1.000
Diabetes	5	5	1.000
Body mass index (kg/m^2^ BSA)	28.7 (26.8, 33.2)	27.6 (25.2, 32.3)	0.339
Laboratory testing			
NT‐proBNP (ng/L)	255 (102, 606)	75 (50, 134)	**<0.001**
Echocardiography			
E/e′ rest	12.5 (9.7, 13.3)	9.15 (7.5, 10.7)	**<0.001**
E/e′ stress^a^	13.8 (10.8, 15.9)	11.0 (10.0, 14.0)	0.120
LAVI (mL/m^2^ BSA)	43.8 (36.6, 54.2)	36.2 (29.2, 41.1)	**0.001**
TAPSE (mm)^a^	24 (21.2, 27.2)	22.5 (20.5, 25.7)	0.335
PAPsys (mmHg)^a^	28 (23.5, 33.1)	22.8 (19.6, 24.7)	**0.001**

*Note*: Categorical parameters are reported in absolute numbers and were compared using the *χ* ^2^ test. Independent continuous parameters are presented as medians with interquartile ranges and were compared by using the Mann–Whitney *U* test. Bold *P* values indicate statistical significance below 0.05. The data shown in this table have been published previously.^21^

^a^ *n*—numbers differ for echocardiographic assessments: TAPSE (*n* = 59), PAPsys (*n* = 56), E/e′ stress (*n* = 50).

Abbreviations: BSA, body surface area; LAVI, left atrial volume index; PAPsys, systolic pulmonary artery pressure; TAPSE, tricuspid annular plane systolic excursion.

HFpEF was defined as PCWP ≥ 15 mmHg at rest and/or ≥25 mmHg during exercise. HFpEF patients diagnosed at rest are referred to as overt HFpEF while patients diagnosed during exercise‐stress only are referred to as masked HFpEF. In the absence of pathological findings on echocardiography, RHC and CMR patients were classified as non‐cardiac dyspnoea (NCD). All analyses were blinded to the classification of patients. All patients gave informed consent before participation. The study was approved by the local ethics committee of the University of Göttingen and conducted according to the tenets of the Declaration of Helsinki.

### CMR‐based quantification of cardiac function

CMR imaging was performed on a clinical 3.0 T Magnetom Skyra MRI scanner (Siemens healthcare, Erlangen, Germany). Balanced steady‐state free precession (bSSFP) cine sequences were used for conventional data acquisition at rest in two‐, three‐ and four‐chamber (Ch) long axis (LAX) as well as short axis (SAX) orientations from base to apex. Standard resolution was as follows: 30 frames per cardiac cycle and a spatial resolution of 1.8 × 1.8 mm in‐plane and 8 mm through‐plane. Post‐processing analyses included RV and LV volumetric assessment as well as FT deformation imaging using commercially available software (2D CPA MR, Cardiac Performance Analysis, TomTec Imaging Systems, Unterschleißheim, Germany). CMR FT included RV global longitudinal strain and RA total strain/reservoir function (Es).^22^, ^23^, ^24^

Real‐time (RT) CMR data acquisition at rest and during exercise was based on bSSFP sequences using a highly undersampled radial encoding scheme, as described previously.^25^ RT‐CMR sequences were acquired for two and four‐Ch LAX views as well as a SAX stack. Standard resolution was as follows: 30 frames per second temporal resolution, 1.6 × 1.6 mm spatial resolution and 6 mm slice thickness. RV/RA long axis strains (LAS) were assessed at rest and during exercise‐stress on four‐Ch views measuring the distance between the middle of a line connecting the origins of the tricuspid leaflets and epicardial apical RV border or most distal wall of the RA, respectively.^26^ Cardiac volumes at rest and during exercise‐stress were assessed on the SAX stack covering an entire cardiac cycle. Volumes assessed included end‐diastolic volume (EDV), end‐systolic volume (ESV) and stroke volume (SV). A CMR‐compatible supine ergometer (Lode, Leiden, the Netherlands) was used for exercise stress. The CMR exercise‐stress protocol was identical to that for RHC/echocardiography.

### Haemodynamic parameters

An elevated end‐systolic pressure (ESP) of the PA for a given SV is indicative of increased resistance and impedance. The ESP/volume ratio (Ea) of the PA can therefore be considered a surrogate marker for afterload.^27^, ^28^

$$\mathrm{Ea}=\frac{\text{Endsystolic pressure}\;\left(\mathrm{ESP}\right)}{\text{Stroke volume}\;\left(\mathrm{SV}\right)}.$$

ESP was calculated using the formula $\mathrm{ESP}=0.98*\left[\mathrm{DBP}+\frac{\mathrm{SBP}-\mathrm{DBP}}{3}\right]+11$ where DBP is the diastolic pulmonary blood pressure and SBP is the systolic pulmonary blood pressure.^28^

The end‐systolic elastance (Ees) is typically defined as the change in pressure that occurs for a given change in volume.^29^ It is defined as the ratio of ESP over ESV^30^ $\mathrm{Ees}=\frac{\mathrm{ESP}}{\mathrm{ESV}}$.

Ventriculo‐arterial coupling was assessed using the ratio of afterload by means of Ea to contractility Ees.

### Statistical analyses

All metric parameters were tested for normal distribution using the Shapiro–Wilk test and are presented as median and interquartile range (IQR). Categorical variables are presented as numbers and corresponding percentages. Differences between two groups were tested by application of the Mann–Whitney *U* or *χ* ^2^ test as appropriate. The Spearman's rank correlation coefficient was used to evaluate correlations. A two‐tailed *P* value <0.05 was considered significant. Analyses were performed using SPSS version 29.0 (IBM, Armonk, NY, USA).

## Results

### Baseline characteristics

The final study population consisted of 68 (HFpEF *n* = 34 and NCD *n* = 34) out of initially 75 recruited patients after excluding 7 patients for novel diagnosis of exclusion criteria on CMR (*n* = 4 myocardial ischaemia, *n* = 1 amyloidosis, *n* = 1 hypertrophic cardiomyopathy and *n* = 1 significant aortic valve stenosis). Baseline characteristics according to HFpEF and NCD have been reported elsewhere.^21^ Briefly, HF patients were older (69 vs. 66 years, *P* = 0.034) and suffered more often from atrial fibrillation [*n* = 16 (47%) vs. *n* = 5 (14.7%), *P* = 0.004]. There were no significant differences in indices for afterload (*P* = 0.079/0.165), contractility (*P* = 0.796/0.763) and coupling (*P* = 0.311/0.622) at rest and during exercise‐stress comparing patients with and without atrial fibrillation. There were no significant differences in cardiovascular risk factors (*P* ≥ 0.339, *Table* 1).

### CMR‐derived haemodynamic parameters

#### Afterload

Afterload increased from rest to stress (1.07 to 2.03, *P* < 0.001). Afterload Ea correlated positively with invasive RHC‐derived PVR at rest (Ea *r* = 0.39, *P* = 0.001) and during exercise‐stress (Ea *r* = 0.47, *P* < 0.001). In contrast, there was a negative association of afterload Ea and CI, which became apparent during exercise‐stress only (rest: Ea *r* = −0.12, *P* = 0.345, exercise‐stress: Ea *r* = −0.29, *P* = 0.019), *Table* 2, which was in line with the negative correlation of PVR and CI (rest: −0.52, *P* < 0.001, stress: −0.65, *P* < 0.001).

**Table 2:** Correlation of CMR and RHC haemodynamic parameters.

Variable	Ea		Ees		Ea/Ees	
	Rest	Stress	Rest	Stress	Rest	Stress
PVR rest	0.39, *P* = 0.001	0.41, *P* < 0.001	0.44, *P* < 0.001	0.44, *P* < 0.001	−0.15, *P* = 0.237	0.02, *P* = 0.856
PVR stress	0.35, *P* = 0.003	0.47, *P* < 0.001	0.44, *P* < 0.001	0.55, *P* < 0.001	−0.16, *P* = 0.199	0.02, *P* = 0.902
CI rest	−0.12, *P* = 0.345	−0.23, *P* = 0.061	−0.21, *P* = 0.082	−0.25, *P* = 0.043	0.16, *P* = 0.188	−0.03, *P* = 0.821
CI stress	−0.35, *P* = 0.004	−0.29, *P* = 0.019	−0.54, *P* < 0.001	−0.41, *P* < 0.001	0.26, *P* = 0.035	0.05, *P* = 0.722
PCWP rest	0.45, *P* < 0.001	0.29, *P* = 0.018	0.54, *P* < 0.001	0.35, *P* = 0.004	−0.15, 0.225	−0.01, *P* = 0.963
PCWP stress	0.32, *P* = 0.008	0.57, *P* < 0.001	0.52, *P* < 0.001	0.56, *P* < 0.001	−0.26, *P* = 0.034	0.11, *P* = 0.395
PA rest	0.63, *P* < 0.001	0.39, *P* = 0.001	0.65, *P* < 0.001	0.42, *P* < 0.001	−0.10, *P* = 0.426	0.03, *P* = 0.812
PA stress	0.40, *P* < 0.001	0.69, *P* < 0.001	0.50, *P* < 0.001	0.68, *P* < 0.001	−0.19, *P* = 0.132	0.12, *P* = 0.327
RV LAS rest	0.27, *P* = 0.029	0.27, *P* = 0.032	0.46, *P* < 0.001	0.46, *P* < 0.001	−0.20, *P* = 0.111	−0.14, *P* = 0.291
RV LAS stress	−0.41, *P* < 0.001	−0.27, *P* = 0.030	−0.36, *P* = 0.003	−0.31, *P* = 0.013	0.07, *P* = 0.563	−0.03, *P* = 0.830

*Note*: Values indicate regression coefficient *r* and associated *P* value. Correlation indices were assessed using Spearman rank correlation coefficients.

Abbreviations: CI, cardiac index; CMR, cardiovascular magnetic resonance; PA, pulmonary artery pressure; PCWP, pulmonary capillary wedge pressure; PVR, pulmonary vascular resistance; RHC, right heart catheterization.

**Figure 1:**
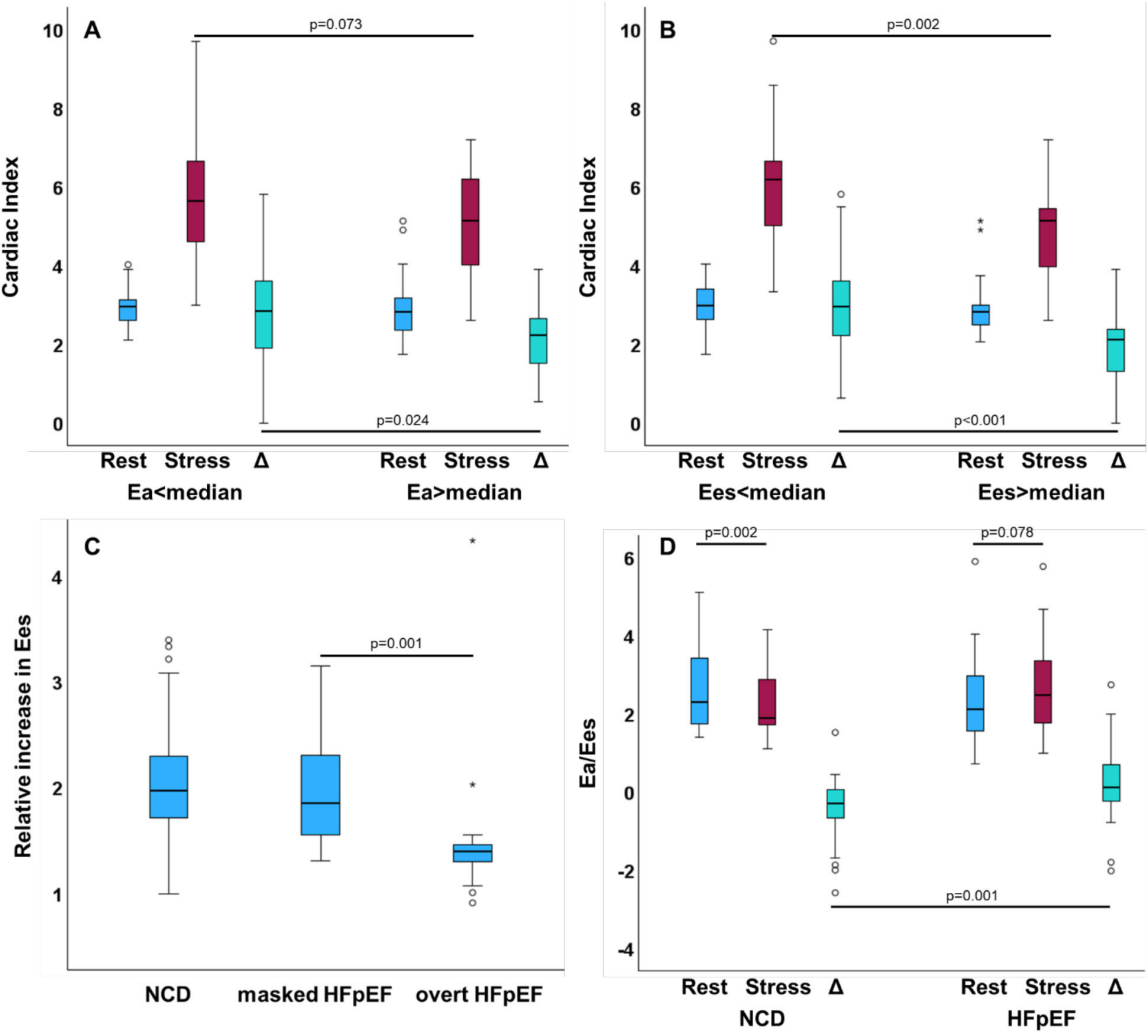
The boxplot shows the median, 1/3 interquartile and 1.5 × IQR whiskers for cardiac index according to (A) afterload Ea or (B) contractility Ees dichotomized at the median at rest and during exercise‐stress with the respective delta accordingly for the overall study population. (C) The relative increase of contractility Ees from rest to stress in non‐cardiac dyspnoea (NCD) as well as masked and overt heart failure with preserved ejection fraction (HFpEF). (D) Right‐ventricular pulmonary artery coupling by the means of afterload Ea to contractility Ees in NCD and HFpEF. Statistics were calculated using the Mann–Whitney *U* test if independent or the Wilcoxon signed‐rank test if dependent.

Dichotomization of resting afterload Ea at the median resulted in similar CI in both groups at rest (below: 2.9 vs. above: 2.8, *P* = 0.552) with a statistical trend towards impaired CI during exercise‐stress in patients with resting Ea above the median (below: 5.6 vs. above: 5.1, *P* = 0.073). This was linked to a significantly impaired capacity to increase CI in patients with resting Ea above compared with below the median (ΔCI 2.2 vs. 2.9, *P* = 0.024, *Figure* 1).

This was paralleled by an increase in RV LAS from rest to exercise‐stress in patients with afterload Ea below the median (22.6 vs. 28.6%, *P* = 0.011) but not in patients with Ea above the median (26.1 vs. 25.7%, *P* = 0.765).

#### Contractility

Similar to afterload Ea, contractility increased from rest to stress (0.46 to 0.88, *P* < 0.001). Contractility Ees correlated positively with PVR at rest (Ees *r* = 0.44, *P* < 0.001) and during exercise‐stress (Ees *r* = 0.55, *P* < 0.001). There was a negative association of contractility Ees with cardiac index and RV LAS, which became apparent during exercise‐stress only (CI rest *r* = −0.21, *P* = 0.082; CI exercise‐stress *r* = −0.37, *P* = 0.002; RV LAS rest *r* = 0.463, *P* < 0.001; RV LAS exercise‐stress *r* = −0.307, *P* = 0.013, *Table* 2).

Dichotomization of resting contractility Ees at the median revealed similar CI in both groups at rest (below: 3.0 vs. above: 2.7, *P* = 0.440), while significant differences emerged during exercise‐stress with reduced CI in patients with Ees above the median at rest (below: 5.8, above: 4.4, *P* = 0.002). This was linked to a significantly impaired capacity to increase CI in patients with resting contractility Ees above compared with below the median (ΔCI 2.1 vs. 2.9, *P* < 0.001, *Figure* 1).

### HFpEF versus NCD

Haemodynamic measurements according to HFpEF and NCD are reported in *Table* 3. Compared with NCD, HFpEF patients showed increased afterload at rest (Ea 1.20 vs. 0.85, *P* = 0.001) and during exercise‐stress (Ea 2.48 vs. 1.53, *P* < 0.001). Comparing rest to exercise‐stress, afterload Ea increased in NCD, masked and overt HFpEF (*P* < 0.001 for all). However, the increase of afterload from rest to stress was higher in HFpEF compared with NCD (1.32 vs. 0.78, *P* = 0.002) in *Table* 3 and numerically highest in masked HFpEF compared with NCD (1.42 vs. 0.78, *P* < 0.001) while not reaching statistical significance compared with overt HFpEF (1.42 vs. 0.81, *P* = 0.174).

**Table 3:** Haemodynamic indices.

Variable	HFpEF	Significance *P* rest vs. stress	NCD	Significance *P* rest vs. stress	Significance *P* HFpEF vs. NCD
Non‐invasive					
Afterload Ea					
Rest	1.20 (0.94, 1.58)	**<0.001**	0.85 (0.66, 1.28)	**<0.001**	**0.001**
Stress	2.48 (1.93, 3.38)		1.53 (1.17, 2.18)		**<0.001**
Δ	1.32 (0.71, 1.87)		0.78 (0.32, 1.16)		**0.002**
Contractility Ees					
Rest	0.61 (0.45, 0.87)	**<0.001**	0.37 (0.31, 0.47)	**<0.001**	**<0.001**
Stress	1.00 (0.83, 1.34)		0.74 (0.59, 0.89)		**<0.001**
Δ	0.38 (0.23, 0.55)		0.35 (0.24, 0.51)		0.634
Coupling (Ea/Ees)					
Rest	2.15 (1.54, 3.04)	0.078	2.31 (1.74, 3.50)	**0.002**	0.114
Stress	2.48 (1.69, 3.39)		1.90 (1.71, 2.91)		0.452
Δ	0.13 (−0.26, 0.82)		−0.28 (−0.67, 0.06)		**0.001**
Invasive					
PVR					
Rest	1.61 (1.22, 2.31)	0.694	1.58 (0.98, 1.83)	0.929	0.164
Stress	1.54 (1.24, 2.40)		1.35 (1.04, 1.94)		0.194
Cardiac index					
Rest	2.9 (2.4, 3.2)	**<0.001**	2.9 (2.6, 3.4)	**<0.001**	0.663
Stress	5.2 (3.7, 6.1)		6.0 (4.8, 6.7)		**0.016**
ΔRest/stress	2.2 (1.3, 2.9)		2.8 (2.1, 3.6)		**0.008**
PCWP					
	13 (11, 18)	**<0.001**	8 (6, 10)	**<0.001**	**<0.001**
Stress	27 (26, 31)		18 (11, 22)		**<0.001**
PA					
Rest	22 (20,28)	**<0.001**	17 (14, 19)	**<0.001**	**<0.001**
Stress	44 (39, 52)		34 (25, 39)		**<0.001**

*Note*: Cardiac index given in L/min/m^2^ body surface area. Continuous parameters are presented as medians with interquartile ranges and were compared using the Mann–Whitney *U* test if independent or the Wilcoxon signed‐rank test if dependent. Bold *P* values indicate statistical significance below 0.05.

Abbreviations: PA, pulmonary artery pressure (mmHg); PCWP, pulmonary capillary wedge pressure (mmHg); PVR, pulmonary vascular resistance (Wood units).

Similarly, compared with NCD, HFpEF patients showed increased contractility at rest (Ees 0.61 vs. 0.37, *P* < 0.001) and during exercise‐stress (Ees 1.00 vs. 0.74, *P* < 0.001). Contractility Ees increased in NCD (*P* < 0.001), masked (*P* < 0.001) and overt HFpEF (*P* = 0.002). However, the relative increase of contractility from rest to stress was smallest in overt HFpEF (overt 1.40 vs. masked 1.86, *P* = 0.001) and highest in NCD (HFpEF 1.56 vs. NCD 1.97, *P* = 0.022).

### Coupling

The out‐of‐proportion increase in afterload compared with contractility in HFpEF is reflected in ventriculo‐arterial coupling. The ratio Ea/Ees decreased in NCD from rest to exercise stress (2.31 vs. 1.90, *P* = 0.002) but not in HFpEF, with a statistical trend towards an increase rather than a decrease (2.15 vs. 2.48, *P* = 0.078).

## Discussion

The results from the present sub‐study of the HFpEF‐Stress trial demonstrate insights into RV–PA interplay in different degrees of post‐capillary PH (NCD, masked and overt HFpEF). The interplay of afterload and contractility is of utmost importance to uphold CO in response to exercise‐stress.

First, elevation of RV afterload and contractility already at rest is associated with impaired functional reserve during exercise‐stress as appreciated from reduced increase in CI and RV LAS. Second, HFpEF patients showed increased afterload and contractility compared with NCD as well as a disproportionate increase in afterload from rest to exercise‐stress. Third, the accompanied increase in contractility from rest to exercise was seen in descending order from NCD to masked and overt HFpEF. Lastly, the out‐of‐proportion increase of afterload to contractility in HFpEF but not NCD is reflected in impaired ventriculo‐arterial coupling (Ea/Ees).

### RV–PA haemodynamic interplay

The out‐of‐proportion increase of PVR in latent PVD correlates negatively with CI.^11^ Caravita et al. discussed dynamic tricuspid regurgitation, altered ventilatory control and pulmonary vascular hyperreactivity during exercise‐stress as underlying causes.^10^

Both RV afterload and contractility increased from rest to exercise‐stress and demonstrated a positive correlation with PVR, which was even stronger during exercise‐stress. As expected, RV afterload demonstrated a negative correlation with CI, which however became apparent during challenged haemodynamic conditions by exercise‐stress only. In a healthy heart, contractility positively correlates to cardiac index, as higher SVs are aligned with increased contractility.^31^ At first glance, the present negative correlation of contractility Ees and CI during exercise‐stress may thus seem counterintuitive. However, reduced CI despite increased RV contractility may be a feature of RV functional failure to overcome afterload, with only inadequately increased contractility resulting in impaired RV–PA coupling during exercise‐stress.

Indeed, patients with resting afterload above the median showed no increase in RV LAS from rest to stress as opposed to patients with afterload laying below the median who showed a compensatory increase. Furthermore, while at rest a positive correlation was observed for RV LAS and afterload as well as contractility, respectively; during exercise, the correlation was found to invert and become negative. This suggests that the right ventricle is unable to provide an adequate compensatory response to elevated afterload during exercise. This is supported by previous data on contractility (Ees) and afterload (Ea) in chronic thromboembolic PH (CTEPH) prior to and following pulmonary endarterectomy (PEA).^13^ Following PEA, the decline in PVR was paralleled by a 62% reduction in afterload Ea, a 28% decrease in contractility Ees and, as a result, a 14% increase in RV EF. While PVR and afterload were elevated prior to pulmonary embolism evacuation, RV contractility Ees was unable to elevate sufficiently to compensate for the distinctly increased PVR, resulting in impaired RV EF.

In line, patients with resting afterload Ea or contractility Ees above the median showed similar CI at rest but smaller increases in CI from rest to stress compared with patients with either index below the median. Consequently, whilst increased RV contractility Ees may be regarded as a compensatory feature to overcome PA afterload Ea or PVR, respectively, particularly in patients with high values at rest (that is already a compensatory mechanism in place), further haemodynamic challenges during exercise‐stress may not be adequately addressed, resulting in exercise‐induced functional failure. This finding is consistent with the results of Caravita et al. in latent PVD.^10^ First, they demonstrated that CI was reduced in patients with concomitant PVD and HFpEF compared with patients with HFpEF alone and that patients with concomitant PVD revealed an inadequate increase in CO during exercise‐stress.^10^ Second, it was shown that patients with higher contractility at rest have a tendency towards lower CI with a subsequent insufficient increase in response to exercise‐stress.^10^

### RV–PA coupling in HFpEF

By definition, HFpEF is associated with post‐capillary PH. HFpEF patients show both increased afterload and contractility at rest compared with NCD. In line with the pathophysiological alterations outlined above, this may indicate an early RV compensatory feature of increased contractility in response to increased afterload in HFpEF. Increased contractility as a compensatory feature for afterload has been previously demonstrated for LV function with elevation of LV Ees in hypertensive patients in the absence of HF as well as HFpEF patients compared with control residents even after adjustment for age, sex, and body size.^32^

Although both afterload and contractility increased from rest to exercise‐stress in HFpEF and NCD, the increase of afterload was distinctly more pronounced in HFpEF as compared with NCD, which was paralleled by a smaller relative increase of contractility in HFpEF. In accordance with the data for the LV,^32^ the RV–PA coupling at rest was similar comparing HFpEF to NCD. However, the out‐of‐proportion increase in afterload over contractility in HFpEF during exercise‐stress was reflected in an unchanged RV–PA coupling index Ea/Ees in HFpEF as opposed to NCD patients, who demonstrated a decrease of the Ea/Ees ratio. This may be indicative of RV functional failure to compensate for afterload in HFpEF. In line, Borlaug et al. demonstrated an increase in CO during exercise limited to a mere 60% rise in HFpEF, whereas in the control group, the rise in CO was 126%.^8^

Intriguingly, early remodelling patterns may not be recognized at rest with masked HFpEF becoming apparent during exercise‐stress testing only (PCWP ≥ 25 mmHg during exercise‐stress but <15 mmHg at rest). Noteworthy, masked HFpEF showed the highest increase in afterload, highlighting the importance of early exercise‐stress induced pathophysiological alterations. Furthermore, the relative increase was smallest in overt HFpEF, followed by masked HFpEF and NCD in ascending order, potentially indicating disease progression.

## Limitation

The HFpEF trial was conducted as a feasibility study for a newly developed imaging technique. The study was designed as a single‐centre study, with all data collected at a single research facility. The patient cohort was highly selective, with great care taken to avoid any potential confounding effects on haemodynamics. In addition, patients with permanent atrial fibrillation were excluded, resulting in a potential selection bias with the exclusion of patients with more severe diastolic dysfunction. Moreover, to calculate Ea and Ees, it was necessary to obtain RV volumes during both rest and exercise. However, RT images tend to underestimate RV volumes in comparison to bSSFP cine sequences, with a subsequent error in calculating Ea and Ees. However, a comparison was only made between Ea and Ees itself, which should minimize the impact on comparisons between the RT cine measurements.

The formula for calculating ESP was derived from left ventricular models. While it has previously been employed for the RV in CTEPH, the present population shows lower PA pressure and therefore applicability may be limited. Because the precise determination of Ees requires simultaneous invasive pressure–volume measurements with acute preload reduction, we use an approximation of Ees to estimate Emax. Ultimately, this is not entirely accurate and only a surrogate evaluation, especially in patients with lower PA pressure, as the volume at zero pressure is not negligible and the pressure conditions do not correspond to those of the left ventricle.^33^ Unfortunately, RHC assessment did not include ESP and thus the non‐invasive surrogate parameter could not be evaluated in its invasive context.

Different subgroups of HFpEF patients have been described in the past years, with patients with LVEF 50%–60% reported to have reduced contractility, increased remodelling as appreciated from higher extracellular volume fraction and impaired ventriculo‐arterial coupling compared with HFpEF with LVEF > 60%.^34^ In line, HFpEF patients with LVEF < 60% show no impact of anti‐inflammatory treatment using Anakinra, as assessed by peak oxygen uptake, as opposed to patients with LVEF > 60%.^35^ Unfortunately, the sample size of the present population is too small to evaluate specific HFpEF subgroups.

## Conclusion

During resting conditions, elevation of contractility may be indicative of increased afterload as seen in HFpEF patients. Both increased afterload and contractility at rest are associated with reduced functional reserve during exercise‐stress. Furthermore, the haemodynamic demands stemming from exercise‐stress lead to an out‐of‐proportion increase of afterload over contractility in HFpEF, resulting in exertional functional failure. As contractility may be increased in HFpEF at rest, more detailed assessments at rest or exercise‐stress testing may be warranted.

## Conflict of interest statement

None disclosed.

## Author contributions

S. J. B. and A. S. designed the study protocol, performed data acquisition, performed statistical analyses and drafted the manuscript. J. S. W. performed statistical analyses and drafted the manuscript. A. S., T. L., S. D. K., S. K. and J. T. K. were involved in data acquisition, and together with J. M. T., A. R., S. S. and G. H. revised the manuscript and participated in the scientific discussion during the study. All authors read and approved the final manuscript.

## Funding

The study was funded by a grant from the German Centre for Cardiovascular Research (DZHK).

## Data Availability

Regarding data availability, we confirm that all relevant data are within the paper and all data underlying the findings are fully available without restriction and can be accessed at the University Medical Centre Göttingen by researchers who meet the criteria for access to confidential data.
